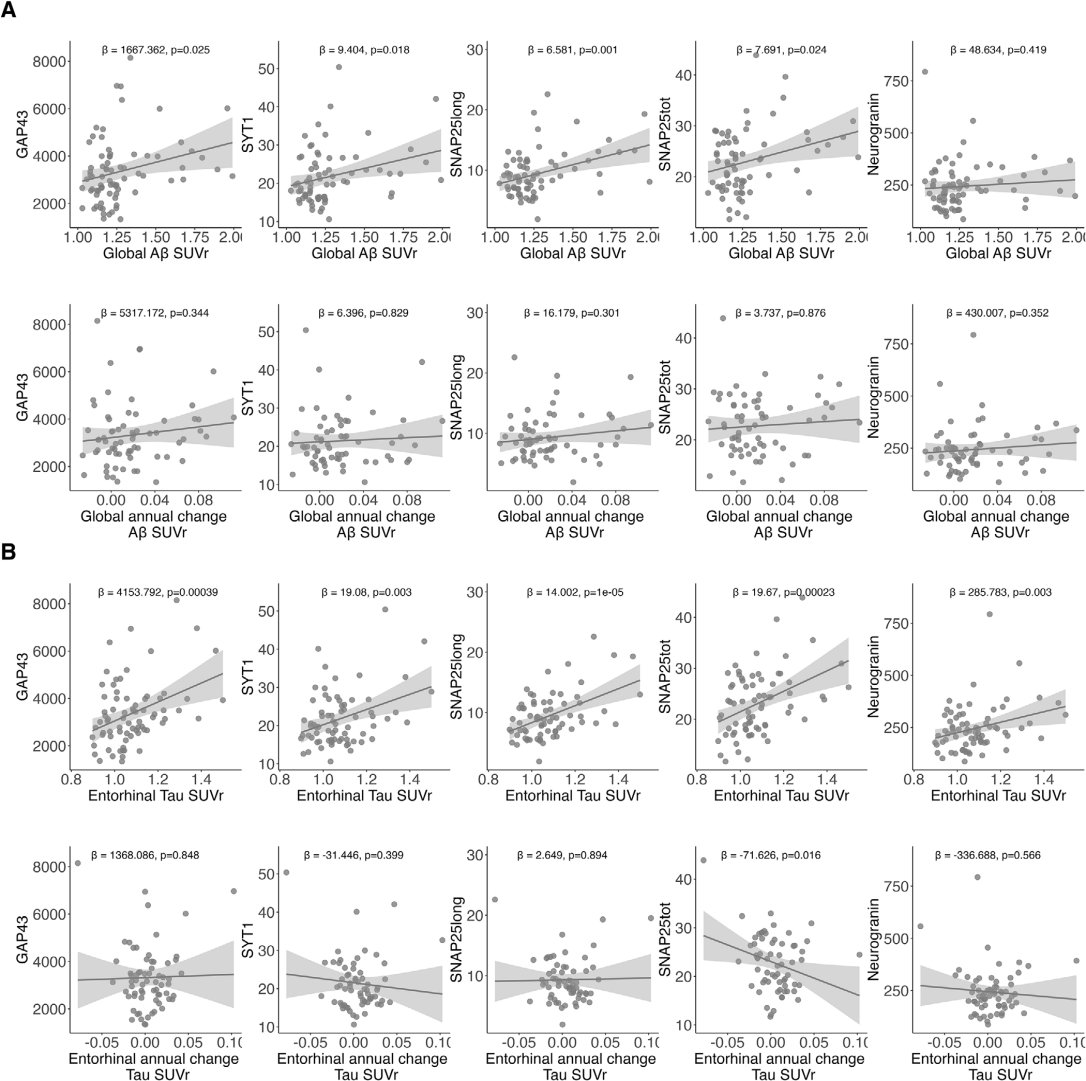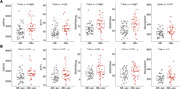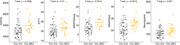# Longitudinal association between synaptic dysfunction and Alzheimer's disease pathology in preclinical Alzheimer's disease

**DOI:** 10.1002/alz.093923

**Published:** 2025-01-09

**Authors:** Valentin Ourry, Alfonso Fajardo Valdez, Jean‐Paul Soucy, Nicholas J. Ashton, Johanna Nilsson, Ann Brinkmalm, Henrik Zetterberg, Kaj Blennow, John C.S. Breitner, Judes Poirier, Sylvia Villeneuve

**Affiliations:** ^1^ Douglas Mental Health University Institute, Centre for Studies on the Prevention of Alzheimer's Disease (StoP‐AD), Montréal, QC Canada; ^2^ Douglas Mental Health University Institute, Montreal, QC Canada; ^3^ McConnell Brain Imaging Centre, Montreal Neurological Institute and Hospital, McGill University, Montreal, QC Canada; ^4^ Centre for Age‐Related Medicine, Stavanger University Hospital, Stavanger Norway; ^5^ Department of Psychiatry and Neurochemistry, Institute of Neuroscience and Physiology, The Sahlgrenska Academy, University of Gothenburg, Mölndal, Gothenburg Sweden; ^6^ Department of Psychiatry and Neurochemistry, Institute of Neuroscience and Physiology, The Sahlgrenska Academy at the University of Gothenburg, Gothenburg Sweden; ^7^ Hong Kong Center for Neurodegenerative Diseases, Hong Kong China; ^8^ Institute of Neuroscience and Physiology, Sahlgrenska Academy at the University of Gothenburg, Göteborg Sweden; ^9^ Department of Psychiatry, McGill University, Montréal, QC Canada; ^10^ Douglas Mental Health Research Centre, Montreal, QC Canada; ^11^ McConnell Brain Imaging Centre ‐ McGill University, Montreal, QC Canada

## Abstract

**Background:**

Synapse loss in Alzheimer’s disease (AD) is correlates closely with cognitive impairment. Recent evidence suggests that synapse loss is promoted by amyloid‐beta, leading in turn to the spread of tau pathology. We sought to assess: 1) the association in positron emission tomography (PET) between several cerebrospinal fluid (CSF) synaptic biomarkers and amyloid and tau burden, as well as their annual change; and 2) the potential clinical utility of these synaptic biomarkers in preclinical AD.

**Methods:**

Mass spectrometry assays quantified cerebrospinal fluid (CSF) markers of synaptic dysfunction (GAP43, SYT1, SNAP25 and Neurogranin) on 75 older adults (age 67.1 ± 4.8 years, 69.3% female) from the PREVENT‐AD cohort. These persons also had longitudinal amyloid ([18F]‐NAV4694) and tau ([18F]‐AV1451) PET scans performed over approximately 4.5 years. All were cognitively unimpaired (CU) at baseline, but 19 developed mild cognitive impairment (MCI) during follow‐up. We examined: 1) the association between these synaptic dysfunction biomarkers and global Aß and entorhinal tau burden as well as their annual change, adjusting for age and sex; and 2) synaptic biomarker differences across those with Aß at baseline vs. those who subsequently developed such pathology, and between individuals who remained CU vs. those who developed MCI during follow‐up. Analyses were considered significant at p=0.05/5=0.01 to account for multiple comparisons.

**Results:**

Amyloid burden was positively associated with increased SNAP25long, while tau burden was associated with all 5 synaptic biomarkers (Figure 1). No association was found between these markers and amyloid and tau annual change. GAP43 and SNAP25 differed in Aß‐ and Aß+ groups but showed no difference across amyloid accumulators’ groups (Figure 2). Finally, MCI individuals had higher SNAP25long and GAP43 concentration than CU individuals (Figure 3).

**Conclusions:**

GAP43 and SNAP25, being markers associated with neuronal growth and synaptic vesicle fusion, appear closely related to amyloid and tau burden, but only at baseline, even in preclinical AD. These biomarkers of synapse loss appear also to have clinical relevance, with differences observed in MCI compared to CU individuals. Future studies should explore these biomarkers, alongside pathology reduction, as potential targets for AD prevention to confirm these preliminary findings.